# LGBTQ College student health and wellbeing at the onset of the pandemic: additional evidence and lessons learned from COVID-19

**DOI:** 10.1186/s12889-023-15909-z

**Published:** 2023-05-26

**Authors:** Gilbert Gonzales, Emilio Loret de Mola, Lee Robertson, Kyle A. Gavulic, Tara McKay

**Affiliations:** 1 Department of Medicine, Health & Society, Department of Health Policy, Program for Public Policy Studies, Vanderbilt University, 2301 Vanderbilt Place PMB #351665, Nashville, TN USA; 2 Department of Community Health Sciences, School of Public Health, Boston University, Boston, MA USA; 3 Department of Psychology, Florida State University, Tallahassee, Florida USA; 4 Yale School of Medicine, New Haven, Connecticut USA; 5 Department of Medicine, Health & Society, Vanderbilt University, Nashville, TN USA

**Keywords:** COVID-19, Pandemic, LGBTQ health, Mental health, Health care access, College students, Young adults

## Abstract

**Background:**

The coronavirus (COVID-19) pandemic has killed more than six million people and disrupted health care systems globally. In the United States alone, more than one million people have died from COVID-19 infections. At the start of the pandemic, nearly all aspects of our lives paused to prevent the spread of the novel coronavirus. Many institutions of higher education transitioned to remote learning and enacted social distancing measures. This study examined the health needs and vulnerabilities of lesbian, gay, bisexual, transgender, queer, and questioning (LGBTQ) college students at the start of the COVID-19 pandemic in the United States.

**Methods:**

We fielded a rapid-response online survey between April and June of 2020. We recruited 578 LGBTQ-identifying college students aged 18 years and older by reaching out to LGBTQ-serving organizations on 254 college campuses and via targeted social media advertising.

**Results:**

Approximately 40% of LGBTQ college students surveyed were dissatisfied with life at the start of the COVID-19 pandemic, and almost all (90%) were concerned that COVID-19 would threaten their mental health. Moreover, about 40% of LGBTQ college students reported unmet mental health needs, and 28% were worried about seeking care during the pandemic because of their LGBTQ identity. One out of four LGBTQ college students had to go back in the closet because of the pandemic, and approximately 40% were concerned about their finances or personal safety during the COVID-19 pandemic. Some of these adverse outcomes were prominent among younger students, Hispanic/Latinx students, and students with unsupportive families or colleges.

**Conclusions:**

Our study adds novel findings to the large body of research demonstrating that LGBTQ college students experienced distress and elevated mental health needs early in the pandemic. Future research should examine the long-term consequences of the pandemic among LGBTQ and other minoritized college students. Public health policymakers, health care providers, and college and university officials should provide LGBTQ students affirming emotional supports and services to ensure their success as the COVID-19 pandemic transitions to endemic.

**Supplementary Information:**

The online version contains supplementary material available at 10.1186/s12889-023-15909-z.

## Background

The novel coronavirus disease (COVID-19) pandemic has led to more than 6 million deaths globally, including more than 1 million deaths in the United States [1]. The pandemic has also indirectly affected the public with lasting economic, social, and trauma-related consequences — which may impact population health for years to come. Schools and institutions of higher education, for instance, experienced substantial disruptions to teaching and learning throughout the pandemic. Starting in March 2020, many colleges and universities suspended their in-person classes, transitioned to online coursework, and closed their on-campus residential halls to reduce the spread of COVID-19. By the end of March 2020, more than one thousand colleges and universities in the United States closed [2] which affected more than 25 million college students [3].

A growing body of research has documented the mental health toll of COVID-19 on college and university students across the United States (regardless of sexual orientation and gender identity). An online survey of 725 full-time college students found that approximately 40% of respondents somewhat agreed or agreed with the statement that they were so anxious about COVID-19 that they “couldn’t pay attention to anything else” [4]. Another study assessing the impact of the COVID-19 pandemic on a cohort of 147 college students in Southern California observed higher levels of stress and sleep disruptions due to the amplification of daily stressors once the “shelter-in-place” orders were in place [5]. Data from the Healthy Minds Study [6], a large (*n* = 36,875) sample of college students from at least 28 universities, found that one-fifth of participants reported symptoms of depression and nearly one-third of participants reported symptoms of anxiety by fall of 2020.

A subpopulation of college and university students who already experienced pre-existing disparities in mental and physical health includes lesbian, gay, bisexual, transgender, queer, and questioning (LGBTQ) students [7, 8]. LGBTQ populations may have also been disproportionally affected by the pandemic due to their higher risks for severe COVID-19 illness, working in industries sensitive to COVID-19 related closures, and persistent barriers to LGBTQ-affirming health care [7, 9–11]. Of particular concern are the LGBTQ college and university students who may have been sent to hostile homes and environments at the start of the COVID-19 pandemic. According to the American College Health Association’s National College Health Assessment, approximately 20% of college students in the spring of 2020 identified as gay, lesbian, bisexual, or other non-heterosexual identities [12].

Several peer-reviewed studies (excluding editorials and commentaries) have examined the health and wellbeing of LGBTQ college students during the COVID-19 pandemic (Table 1). Most prior studies were recruited and conducted online and early in the pandemic (April through August of 2020). Nine studies [13–21] directly assessed levels of psychological distress, anxiety, and depression among LGBTQ college students during the pandemic. Members of our team (Gonzales et al.) [13] previously reported that 60% of LGBTQ college students were living with frequent mental distress, depression, and/or anxiety when college and universities went remote or virtual in the first wave of COVID-19. LGBTQ college students residing at their parental home during the COVID-19 pandemic were more likely to experience identity concealment, familial rejection, and fewer opportunities for expressing their sexual orientation or gender identity [22, 23], some of which may have led to even higher levels of psychological distress [24]. Zhang et al. [19] found that having parents aware and supportive of the student’s LGBTQ status was associated with better wellbeing than not having LGBTQ-supportive parents. Salerno et al. [16, 17] also found that LGBTQ college students reporting increased alcohol use, and LGBTQ college students experiencing LGBTQ identity-based discrimination or harassment were more likely to report psychological distress. Beyond mental health and substance use, only one study has monitored behaviors to prevent the spread of COVID-19. Lawrence et al. [25] found that most LGBTQ college students stayed home when needed (except when seeking essential needs), avoided social gatherings, maintained six feet distancing, and routinely wore face masks.

**Table 1:** Summary of peer-reviewed studies on LGBTQ college students and their wellbeing during the COVID-19 pandemic

Author (Year)	Title	Sample Size (n)	Data Collection Period	Type of Study	Main Findings
Algarin et al. (2022) [22]	Associations Between Living Arrangement and Sexual and Gender Minority Stressors Among University Students Since the Start of the COVID-19 Pandemic	478 sexual and gender minority college students	May to August 2020	Online survey	Returning to parental home due to COVID-19 was associated with identity concealment and familial rejection
Cerezo et al. (2021) [26]	Understanding the Power of Social Media During COVID-19: Forming Social Norms for Drinking among Sexual Minority Gender Expansive College Women	28 Sexual minority and gender expansive college women	February 11 to May 25, 2020	Online focus groups	Social drinking on social networking platforms increased as a way to address social isolation and pandemic-related stress
Freibott et al. (2022) [20]	The Influence of Race, Sexual Orientation and Gender Identity on Mental Health, Substance Use, and Academic Persistence During the COVID-19 Pandemic	146,810 undergraduate students	Fall 2017 to Winter 2020 (pre-COVID) March to December 2020 (during COVID)	Healthy Minds Study	Sexual and gender minority undergraduate students were more likely to screen for anxiety and/or depression compared to cisgender-heterosexual peers
Gattamorta et al. (2022) [24]	Family Rejection during COVID-19: Effects on Sexual and Gender Minority Stress and Mental Health among LGBTQ University Students	565 sexual and gender minority university students	May 27 to August 14, 2020	Online survey	Familial rejection among sexual and gender minority university students was associated with moderate to severe psychological distress
Gonzales et al. (2020) [13]	Mental Health Needs Among Lesbian, Gay, Bisexual, and Transgender College Students During the COVID-19 Pandemic	477 LGBTQ college students	April 24 to June 5, 2020	Online survey	Approximately 60% of LGBTQ college students reported symptoms of psychological distress, anxiety, and/or depression
Hanna-Walker et al. (2023) [23]	“It’s like and elephant in the room with my family”: LGBTQ + College Students’ Identity Expression During the COVID-19 Pandemic	411 LGBTQ college students	April 29 to May 25, 2020	Online survey	Most LGBTQ + college students perceived restricted opportunities for expressing their sexual orientation and/or gender identity, especially when living with family
Hoyt et al. (2021) [21]	“Constant Stress Has Become the New Normal”: Stress and Anxiety Among U.S. College Students in the Time of COVID-19	707 full-time college students	April 25 to July 31, 2020	Online survey with follow-up	Sexual and gender minority students reported more perceived stress and anxiety than cisgender-heterosexual peers
Hunt et al. (2021) [14]	Gender Diverse College Students Exhibit Higher Psychological Distress Than Male and Female Peers During the Novel Coronavirus (COVID-19) Pandemic	83 gender diverse participants matched with 83 males and 83 females	March 18 to April 1, 2020	Survey sent by email at a public 4-year university	Approximately half of gender diverse college students reported severe psychological distress, which was higher than male and female participants
Lawrence et al. (2021) [25]	LGBTQ + College Students’ Engagement in COVID-Protective and COVID-Risk Behaviors	438 LGBTQ college students	April 29 to May 25, 2020	Online survey	Most LGBTQ college students stayed home except when seeking essential needs, avoided social gatherings, maintained six feet distancing, and wore face masks
Parchem et al. (2021) [15]	Comparison of Anxiety and Depression Rates Among LGBTQ College Students Before and During the COVID-19 Pandemic	3,484 LGBTQ college students sampled before the pandemic and 1,647 sampled mid-pandemic	Spring 2019 semester and Spring 2021 semester	Healthy Minds Study	Among LGBTQ college students, anxiety symptoms were significantly lower during the pandemic than they were pre-pandemic, and there were no changes in depression
Salerno et al. (2021) [16]	Changes in Alcohol Use Since the Onset of COVID-19 are Associated with Psychological Distress Among Sexual and Gender Minority University Students in the U.S	509 LGBTQ college students	May 27, 2020 to August 14, 2020	Online survey	Mean psychological distress was high and 32% of LGBTQ college students reported increased alcohol use since the start of the pandemic
Salerno et al. (2022) [17]	LGBTQ Identity-Related Victimization During COVID-19 Is Associated with Moderate to Severe Psychological Distress Among Young Adults	565 LGBTQ college students	May 27, 2020 to August 14, 2020	Online survey	Past year LGBTQ identity-based discrimination since the start of COVID-19 was associated with greater psychological distress
Wood et al. (2022) [18]	Perceived Impact of COVID-19 on Sexual Health and Access to Sexual Health Services Among University Students in Canada	1,504 university students	December 2020 to January 2021	Online survey	Sexual minority students reported higher levels of stress compared to their heterosexual peers
Zhang et al. (2022) [19]	How Social Support and Parent–Child Relationship Quality Relate to LGBTQ + College Students’ Well-Being During COVID-19	366 LGBTQ college students	April 29, 2020 to May 25, 2020	Online survey	LGBTQ students with more social and familial supports reported better wellbeing than LGBTQ peers without supportive families during the pandemic

The current study provides additional evidence to the array of health disparities experienced by LGBTQ college and university students during the COVID-19 pandemic. In particular, this study examines self-reported health status, access to medical and mental health care, and other concerns related to identity, finances, and personal safety among LGBTQ college students during the COVID-19 pandemic. We also provide novel subgroup and intersectional analyses to identify the LGBTQ subpopulations at greatest risk of COVID-19 vulnerabilities in order to inform tailored interventions.

## Methods

### Study sample

We recruited 578 LGBTQ college students aged 18 years and older to participate in a rapid-response online survey on their health and wellbeing during the COVID-19 pandemic between April 24 and June 5, 2020. Participants were recruited through email listservs, outreach to LGBTQ student organizations, emails sent directly to LGBTQ office directors and/or diversity officers at 254 colleges and universities, and targeted social media advertisements via Facebook and Instagram. Participants resided in 47 states and Puerto Rico. Participants were asked detailed questions about their demographic characteristics, mental and emotional health, access to care, social supports, and socioeconomic vulnerabilities during the COVID-19 pandemic.

### Study outcomes

Among our study sample, we estimated the prevalence of various health outcomes, access to health care, and COVID-19 vulnerabilities experienced by LGBTQ college students. First, we classified whether participants indicated their health as poor or fair health versus excellent, very good, or good health. Then, we created dichotomous outcomes on whether LGBTQ students were dissatisfied with life (versus satisfied with life) and whether they agreed (versus disagreed) that the COVID-19 pandemic threatens their mental health. Next, we measured three dimensions of health care access. We assessed whether LGBTQ students were unable to receive medical care or unable to receive mental health care because of cost, stay-at-home order restrictions, or other reasons (multiple options could have been selected). LGBTQ college students were also asked the extent that they were worried about seeking health care because of their LGBTQ status should they become infected with COVID-19 (a great deal or fair amount versus not much or not at all). Finally, we examined whether LGBTQ college students reported that they have gone back into the closet to some extent because of the COVID-19pandemic, whether the COVID-19 pandemic made participants concerned about their personal finances, or if the COVID-19 pandemic put them in a position where they were concerned about their personal safety.

### Statistical analysis

First, we used descriptive statistics to characterize the study sample. We examined the following sociodemographic characteristics of our sample of LGBTQ college students: sexual orientation (gay/lesbian, bisexual, queer, asexual, questioning, other), gender identity (cisgender man, cisgender woman, transgender, non-binary, gender non-conforming, agender, genderqueer, other), age in years (18–20, 21–24, 25–29, 30 or older), race/ethnicity (White, Black or African American, Hispanic or Latinx, Asian or Pacific Islander, other/multiple races or ethnicities). We also gauged the extent that participant lives were disrupted by COVID-19 (not at all or not much, a fair amount, a great deal), their risk aversions and concerns about COVID-19 (not concerned or moderately concerned, very or extremely concerned), and whether the participant, a family member, or a close friend tested positive for COVID-19. Finally, we examined the following two social supports which may buffer adverse experiences during the COVID-19 pandemic: whether the participant’s immediate family (i.e., parents and siblings) were supportive of their LGBTQ identity, and whether the participant felt supported by their college or university’s LGBTQ office during the COVID-19 pandemic.

After describing the sample, we estimated fully adjusted multivariable logistic regression models to identify the subpopulations at greatest risk for each outcome of interest. All logistic regression models controlled for sexual orientation, gender identity, age, race/ethnicity, experiences and concerns with COVID-19, and social supports from the participant’s family and college. All logistic regression results are presented as adjusted odds ratios (aOR) with 95% confidence intervals (CI). This study was deemed exempt from full review by the Vanderbilt University Institutional Review Board.

## Results

### Demographic characteristics and social supports

Table 2 presents descriptive statistics of sampled LGBTQ college students aged 18 years and older. More than half of the study sample identified as gay, lesbian, bisexual, or queer. Approximately 14% identified as pansexual; 11% identified as asexual; and 14% identified as questioning or other sexual orientations (e.g., demisexual, polysexual, aromantic, etc.). Most sampled LGBTQ college students were cisgender men (17%) or cisgender women (41.2%). The remaining LGBTQ college students identified as non-binary (11.4%), genderqueer (9.2%), transgender (8.5%), gender non-conforming (5%), agender (3.8%), or another gender identity (4%). Most LGBTQ college students were aged 18–24 years (78%). About 63% of the study sample were White, 13% were Hispanic/Latinx, 13% were Asian or Pacific Islander, and 9% were Black or African American. Most (87%) LGBTQ college students reported that their lives were disrupted a fair amount or a great deal by COVID-19, and more than half were very or extremely concerned about COVID-19. Approximately 44% of LGBTQ college students were from families that were unsupportive or did not know about the participant’s LGBTQ identity. A plurality (48%) of LGBTQ college students felt supported by their college or university LGBTQ office, but 31% did not feel supported by their institution’s LGBTQ office.

**Table 2:** Descriptive statistics of LGBTQ college students, 18 years and older

	Sample Size (n)	Percent (%)
**Sexual Orientation**		
Gay/Lesbian	125	21.6
Bisexual	97	16.8
Queer	133	23.0
Pansexual	80	13.8
Asexual	63	10.9
Questioning	39	6.8
Other	41	7.1
**Gender Identity**		
Cisgender Man	98	17.0
Cisgender Woman	238	41.2
Transgender	49	8.5
Non-Binary	66	11.4
Gender Non-Conforming	29	5.0
Agender	22	3.8
Genderqueer	53	9.2
Other	23	4.0
**Age, in years**		
18–20	240	41.5
21–24	211	36.5
25–29	88	15.2
≥ 30	39	6.8
**Race/Ethnicity**		
White	362	62.6
Black or African American	53	9.2
Hispanic/Latinx	76	13.2
Asian or Pacific Islander	74	12.8
Other	13	2.3
**Life Disrupted by COVID-19**		
Not at all or not much	76	13.2
A fair amount	282	48.8
A great deal	220	38.1
**Concerned About COVID-19**		
Not concerned or moderately concerned	264	45.7
Very or extremely concerned	314	54.3
**Participant, family or friend tested positive for COVID-19**		
No	475	82.2
Yes	103	17.8
**Immediately Family Supportive of Participant's LGBTQ Identity**		
Supportive	232	40.1
Neither supportive nor unsupportive	93	16.1
Unsupportive or family does not know	253	43.8
**Feels Supported by their College or University LGBTQ Office**		
Yes	275	47.6
No, not much or no support	178	30.8
No opinion or missing data	125	21.6

Source: Online sample of LGBTQ identified college students from April 24—June 5, 2020

### Health status and access to care

Table 3 presents the prevalence and adjusted odds ratios of self-rated health and mental health concerns among LGBTQ college and university students. Approximately 21% of sampled students reported their health as poor or fair. After controlling for sociodemographic characteristics and social supports, LGBTQ students questioning their sexual orientation (aOR = 3.40; 95% CI = 1.40–8.26) were more likely to report poor/fair health relative to gay/lesbian students. Meanwhile cisgender women (aOR = 3.09; 95% CI = 1.23–7.74), transgender (aOR = 4.09; 95% CI = 1.39–12.04), non-binary (aOR = 4.84; 95% CI = 1.69–13.82), gender non-conforming (aOR = 5.10; 95% CI = 1.49–17.46), agender (aOR = 12.56; 95% CI = 3.58–44.03), genderqueer (aOR = 5.56; 95% CI = 1.89–16.34), and other gender minority (aOR = 4.53; 95% CI = 1.17–17.53) students were more likely to report poor/fair health compared to cisgender men. Relative to White students, Hispanic/Latinx students (aOR = 2.43; 95% CI = 1.32–4.47) were more likely to report poor/fair health. Compared to students with families supportive of their LGBTQ identity, LGBTQ students with unsupportive families or families that did not know of their LGBTQ status (aOR = 1.81; 95% CI = 1.08–3.03) were more likely to self-report poor/fair health.

**Table 3:** Prevalence and adjusted odds ratios of self-rated health and mental health among LGBTQ college students, 18 years old

	**Poor/Fair Health**		**Dissatisfied with Life**		**COVID-19 Threatens Mental Health**	
	Prevalence (%)	aOR (95% CI)	Prevalence (%)	aOR (95% CI)	Prevalence (%)	aOR (95% CI)
**All Sampled LGBTQ College Students**	20.8	N/A	39.8	N/A	90.1	N/A
**Sexual Orientation**						
Gay/Lesbian	13.6	1.00 [Reference]	31.2	1.00 [Reference]	87.2	1.00 [Reference]
Bisexual	10.3	0.51 (0.21–1.25)	41.2	1.28 (0.68–2.41)	86.6	0.86 (0.33–2.28)
Queer	21.1	1.21 (0.58–2.52)	37.6	1.25 (0.69–2.26)	93.2	1.68 (0.62–4.56)
Pansexual	27.5	1.41 (0.64–3.09)	53.8	2.18 (1.13–4.22)**	92.5	1.38 (0.44–4.27)
Asexual	22.2	1.05 (0.43–2.53)	31.8	0.73 (0.35–1.53)	96.8	4.91 (0.90–26.86)
Questioning	41.0	3.40 (1.40–8.26)**	48.7	1.53 (0.68–3.44)	92.3	1.33 (0.30–5.90)
Other	31.7	2.00 (0.77–5.17)	46.3	1.44 (0.63–3.28)	80.5	0.64 (0.21–2.02)
**Gender Identity**						
Cisgender Man	7.1	1.00 [Reference]	29.6	1.00 [Reference]	82.7	1.00 [Reference]
Cisgender Woman	17.7	3.09 (1.23–7.74)**	38.7	0.91 (0.50–1.64)	89.9	1.33 (0.58–3.07)
Transgender	26.5	4.09 (1.39–12.04)**	44.9	1.24 (0.56–2.75)	95.9	3.30 (0.63–17.40)
Non-Binary	27.3	4.84 (1.69–13.82)**	40.9	1.04 (0.49–2.22)	92.4	1.12 (0.33–3.81)
Gender Non-Conforming	27.6	5.10 (1.49–17.46)**	44.8	1.30 (0.49–3.43)	93.1	0.97 (0.17–5.41)
Agender	50.0	12.56 (3.58–44.03)**	50.0	1.49 (0.52–4.29)	90.9	1.16 (0.19–7.33)
Genderqueer	28.3	5.56 (1.89–16.34)**	47.2	1.55 (0.70–3.41)	98.1	5.74 (0.66–49.94)
Other	26.1	4.53 (1.17–17.53)**	47.8	1.27 (0.44–3.68)	82.6	0.78 (0.18–3.36)
**Age, in years**						
18–20	22.1	1.08 (0.55–2.11)	47.1	2.35 (1.32–4.18)**	90.8	1.03 (0.40–2.65)
21–24	20.4	1.10 (0.55–2.18)	37.9	1.51 (0.85–2.70)	89.1	0.85 (0.33–2.19)
25–29	18.2	1.00 [Reference]	28.4	1.00 [Reference]	90.9	1.00 [Reference]
≥ 30	20.5	0.98 (0.35–2.75)	30.8	1.05 (0.43–2.54)	89.7	0.62 (0.15–2.55)
**Race/Ethnicity**						
White	19.3	1.00 [Reference]	38.4	1.00 [Reference]	90.9	1.00 [Reference]
Black or African American	22.6	0.84 (0.39–1.83)	54.7	1.41 (0.73–2.70)	88.7	0.82 (0.29–2.35)
Hispanic/Latinx	34.2	2.43 (1.32–4.47)**	43.4	1.06 (0.61–1.86)	89.5	0.92 (0.36–2.37)
Asian or Pacific Islander	13.5	0.52 (0.23–1.18)	33.8	0.64 (0.35–1.15)	87.8	0.54 (0.22–1.35)
Other	15.4	0.62 (0.12–3.31)	30.8	0.54 (0.15–1.96)	92.3	1.19 (0.13–10.89)
**Life Disrupted by COVID-19**						
Not at all or not much	17.1	1.00 [Reference]	32.9	1.00 [Reference]	76.3	1.00 [Reference]
A fair amount	20.6	1.11 (0.54–2.29)	38.3	1.15 (0.64–2.07)	88.7	2.26 (1.10–4.63)**
A great deal	22.3	1.11 (0.53–2.36)	44.1	1.42 (0.78–2.62)	96.8	7.44 (2.76–20.05)**
**Concerned About COVID-19**						
Not concerned or moderately concerned	18.9	1.00 [Reference]	38.6	1.00 [Reference]	84.9	1.00 [Reference]
Very or extremely concerned	22.3	1.17 (0.74–1.85)	40.8	1.11 (0.76–1.61)	94.6	2.83 (1.47–5.42)**
**Participant, family or friend tested positive for COVID-19**						
No	19.8	1.00 [Reference]	37.9	1.00 [Reference]	89.9	1.00 [Reference]
Yes	25.2	1.35 (0.77–2.34)	48.5	1.50 (0.94–2.40)	91.3	1.34 (0.59–3.09)
**Immediately Family Supportive of Participant's LGBTQ Identity**						
Supportive	15.1	1.00 [Reference]	26.3	1.00 [Reference]	88.4	1.00 [Reference]
Neither supportive or unsupportive	21.5	1.30 (0.67–2.54)	40.9	1.72 (1.00–2.95)**	89.3	1.09 (0.45–2.63)
Unsupportive or family does not know	25.7	1.81 (1.08–3.03)**	51.8	2.96 (1.93–4.53)**	92.1	1.55 (0.76–3.16)
**Feels Supported by their College or University LGBTQ Office**						
Yes	20.0	1.00 [Reference]	34.9	1.00 [Reference]	91.3	1.00 [Reference]
No, not much or no support	23.6	1.23 (0.74–2.04)	43.3	1.45 (0.94–2.23)	92.7	0.92 (0.42–1.99)
No opinion or missing data	18.4	0.77 (0.42–1.42)	45.6	1.69 (1.04–2.76)**	84.0	0.40 (0.19–0.83)**

Source: Online sample of LGBTQ identified college students from April 24—June 5, 2020. Notes: aORs were obtained from multivariable logistic regression models adjusting for all the covariates listed in the table. **p < 0.05

Approximately 40% of LGBTQ college students were dissatisfied with life, and this prevalence was significantly higher for younger LGBTQ college students aged 18–20 years (aOR = 2.35; 95% CI = 1.32–4.18) compared to older LGBTQ college students aged 25–29 years. LGBTQ students with families that were unsupportive or unaware (aOR = 2.96; 95% CI = 1.93–4.53) or neutral (aOR = 1.72; 95% CI = 1.00–2.95) about their LGBTQ status were also more likely to be dissatisfied with life compared to LGBTQ college students with supportive families. Almost all (90%) LGBTQ college students agreed that COVID-19 posed a threat to their mental health, and these concerns were elevated for LGBTQ college students who indicated that COVID-19 disrupted their life by a fair amount (aOR = 2.26; 95% CI = 1.10–4.63) or a great deal (aOR = 7.44; 95%CI = 2.76–20.05) compared to those whose lives were not disrupted by COVID-19.

Table 4 presents the prevalence and adjusted odds ratios of barriers to care experienced by LGBTQ college students at the beginning of the pandemic. Approximately 21% of LGBTQ college students reported unmet medical care needs during the COVID-19 pandemic. Compared to cisgender men, unmet medical care needs were significantly greater for transgender (aOR = 2.91; 95% CI = 1.17–7.22), non-binary (aOR = 2.60; 95% CI = 1.07–6.29), agender (aOR = 3.25; 95%CI = 1.04–10.20), and other gender minority (aOR = 3.41; 95% CI = 1.05–11.05) students. Black or African American students (aOR = 2.12; 95% CI = 1.03–4.38), Hispanic/Latinx students (aOR = 2.43; 95% CI = 1.32–4.47) and LGBTQ college students not supported by their college or university LGBTQ office (aOR = 1.92; 95% CI = 1.16–3.17) were also more likely to report unmet medical care needs compared to White students and students feeling supported by their institution’s LGBTQ office, respectively.

**Table 4:** Prevalence and adjusted odds ratios of barriers to care among LGBTQ college students, 18 years and older

	**Unmet Medical Care**		**Unmet Mental Health Care**		**Worried About Seeking Care Because of LGBTQ Status**	
	Prevalence (%)	aOR (95% CI)	Prevalence (%)	aOR (95% CI)	Prevalence (%)	aOR (95% CI)
**All Sampled LGBTQ College Students**	20.6	N/A	39.5	N/A	28.3	N/A
**Sexual Orientation**						
Gay/Lesbian	18.4	1.00 [Reference]	33.6	1.00 [Reference]	23.3	1.00 [Reference]
Bisexual	13.4	0.71 (0.31–1.60)	42.3	1.15 (0.62–2.16)	20.5	0.80 (0.36–1.78)
Queer	18.8	0.98 (0.49–1.98)	35.3	0.95 (0.53–1.70)	32.3	1.12 (0.56–2.22)
Pansexual	28.8	1.62 (0.77–3.42)	45.0	1.32 (0.69–2.55)	31.1	0.87 (0.40–1.91)
Asexual	25.4	1.37 (0.60–3.13)	47.6	1.46 (0.72–2.96)	28.1	0.75 (0.31–1.81)
Questioning	23.1	1.05 (0.40–2.76)	35.9	0.89 (0.39–2.05)	30.3	0.72 (0.26–1.98)
Other	24.4	0.89 (0.34–2.30)	43.9	1.58 (0.69–3.61)	45.2	1.07 (0.38–3.03)
**Gender Identity**						
Cisgender Man	15.3	1.00 [Reference]	27.6	1.00 [Reference]	12.5	1.00 [Reference]
Cisgender Woman	16.4	1.25 (0.60–2.59)	40.8	1.58 (0.87–2.86)	15.4	1.48 (0.66–3.33)
Transgender	32.7	2.91 (1.17–7.22)**	38.8	1.44 (0.64–3.24)	68.2	21.66 (7.96–58.92)**
Non-Binary	27.3	2.60 (1.07–6.29)**	37.9	1.48 (0.69–3.18)	47.7	7.47 (3.01–18.54)**
Gender Non-Conforming	27.6	2.07 (0.69–6.20)	55.2	3.11 (1.20–8.06)**	34.6	3.88 (1.24–12.18)**
Agender	36.4	3.25 (1.04–10.20)**	59.1	2.63 (0.90–7.69)	60.0	15.13 (4.40–52.01)**
Genderqueer	15.1	1.05 (0.37–2.92)	45.3	2.28 (1.03–5.05)**	28.9	3.22 (1.22–8.53)**
Other	30.4	3.41 (1.05–11.05)**	30.4	1.05 (0.35–3.16)	42.9	7.69 (2.22–26.67)**
**Age, in years**						
18–20	17.9	0.44 (0.24–0.81)**	39.2	1.08 (0.62–1.89)	28.4	0.73 (0.39–1.39)
21–24	20.9	0.56 (0.30–1.03)	45.0	1.44 (0.82–2.50)	26.8	0.70 (0.36–1.34)
25–29	29.6	1.00 [Reference]	33.0	1.00 [Reference]	30.2	1.00 [Reference]
≥ 30	15.4	0.34 (0.12–0.96)**	25.6	0.62 (0.25–1.54)	30.6	0.94 (0.36–2.45)
**Race/Ethnicity**						
White	17.4	1.00 [Reference]	35.6	1.00 [Reference]	25.3	1.00 [Reference]
Black or African American	28.3	2.12 (1.03–4.38)**	45.3	1.16 (0.61–2.19)	28.6	1.18 (0.55–2.56)
Hispanic/Latinx	30.3	2.48 (1.33–4.61)**	46.1	1.45 (0.83–2.51)	37.3	2.18 (1.13–4.21)**
Asian or Pacific Islander	18.9	1.49 (0.73–3.03)	47.3	1.43 (0.82–2.52)	30.0	1.65 (0.84–3.26)
Other	30.8	2.26 (0.60–8.48)	38.5	1.07 (0.30–3.81)	50.0	3.38 (0.85–13.48)
**Life Disrupted by COVID-19**						
Not at all or not much	19.7	1.00 [Reference]	36.8	1.00 [Reference]	22.1	1.00 [Reference]
A fair amount	21.3	1.10 (0.56–2.19)	38.3	0.96 (0.54–1.70)	25.4	1.01 (0.49–2.08)
A great deal	20.0	0.90 (0.44–1.86)	41.8	1.11 (0.62–2.03)	34.2	1.35 (0.64–2.84)
**Concerned About COVID-19**						
Not concerned or moderately concerned	17.4	1.00 [Reference]	40.9	1.00 [Reference]	23.9	1.00 [Reference]
Very or extremely concerned	23.3	1.41 (0.90–2.23)	38.2	0.85 (0.59–1.23)	32.1	1.39 (0.89–2.18)
**Participant, family or friend tested positive for COVID-19**						
No	19.4	1.00 [Reference]	36.6	1.00 [Reference]	27.8	1.00 [Reference]
Yes	26.2	1.39 (0.82–2.38)	52.4	1.66 (1.05–2.63)**	30.5	1.30 (0.74–2.29)
**Immediately Family Supportive of Participant's LGBTQ Identity**						
Supportive	16.8	1.00 [Reference]	26.3	1.00 [Reference]	27.1	1.00 [Reference]
Neither supportive nor unsupportive	25.8	1.48 (0.79–2.78)	52.7	2.84 (1.67–4.84)**	23.3	0.66 (0.33–1.30)
Unsupportive or family does not know	22.1	1.17 (0.71–1.96)	46.6	2.05 (1.35–3.12)**	31.3	1.20 (0.73–1.98)
**Feels Supported by their College or University LGBTQ Office**						
Yes	16.7	1.00 [Reference]	36.7	1.00 [Reference]	29.4	1.00 [Reference]
No, not much or no support	25.8	1.92 (1.16–3.17)**	46.6	1.63 (1.07–2.50)**	30.5	1.02 (0.62–1.68)
No opinion or missing data	21.6	1.44 (0.79–2.62)	35.2	0.93 (0.57–1.53)	21.4	0.64 (0.33–1.24)

Source: Online sample of LGBTQ identified college students from April 24—June 5, 2020. Notes: aORs were obtained from multivariable logistic regression models adjusting for all the covariates listed in the table. **p < 0.05

Nearly 40% of LGBTQ college students reported unmet mental health care needs. The odds of unmet mental health care needs were significantly higher for gender non-conforming students (aOR = 3.11; 95% CI = 1.20–8.06, compared to cisgender men), genderqueer students (aOR = 2.28; 95% CI = 1.03–5.05, compared to cisgender men), LGBTQ college students who knew someone that had tested positive for COVID-19 (aOR = 1.66; 95% CI = 1.05–2.63, compared to LGBTQ students who did not know someone who tested positive for COVID-19), LGBTQ college students with families that were neutral (aOR = 2.84; 95% CI = 1.67–4.84, compared to supportive families) or unsupportive (aOR = 2.05; 95% CI = 1.35–3.12, compared to supportive families) of the participant’s LGBTQ status, and those not feeling enough support from their college or university LGBTQ office (aOR = 1.63; 95% CI = 1.07–2.50, compared to those who felt supported by their institution’s LGBTQ office).

Approximately 28% of LGBTQ college students were worried about seeking care at the start of the pandemic because of their sexual orientation or gender identity. These concerns were significantly higher for LGBTQ college students who identified as transgender (aOR = 21.66; 95% CI = 7.96–58.92), non-binary (aOR = 7.47; 95% CI = 3.01–18.54), gender non-conforming (aOR = 3.88; 95% CI = 1.24–12.18), agender (aOR = 15.13; 95% CI = 4.40–52.01), genderqueer (aOR = 3.22; 95% CI = 1.22–8.53), or other gender-diverse identities (aOR = 7.69; 95% CI = 2.22–26.67) compared to cisgender men. Hispanic and Latinx students (aOR = 2.18; 95%CI = 1.13–4.21) also expressed elevated concerns about seeking care because of their LGBTQ identity compared to their White peers.

### COVID-19 Vulnerabilities

Table 5 presents the prevalence and adjusted odds ratios of COVID-19 vulnerabilities experienced by LGBTQ college students at the beginning of the pandemic. Approximately 1 in 4 LGBTQ college students went “back into the closet” because of the COVID-19 pandemic. Those more likely to go back into the closet included agender students (aOR = 5.27; 95% CI = 1.37–20.26, compared to cisgender men), younger LGBTQ college students aged 18–20 years (aOR = 6.83; 95%CI = 2.74–17.03, compared to 25–29 years) or 21–24 years (aOR = 3.22; 95% CI = 1.29–8.04, compared to 25–29 years) and those with families who were neutral (aOR = 4.39; 95% CI = 1.98–9.72, compared to those who had supportive families) or unsupportive of their LGBTQ status (aOR = 11.16; 95% CI = 5.76–21.63, compared to those who had supportive families). Nearly 40% of LGBTQ college students were facing personal financial hardships during the COVID-19 pandemic, and these concerns were significantly greater for genderqueer students (aOR = 2.85; 95% CI = 1.30–6.22) compared to students who identified as cisgender men. Concerns about personal finances were also greater for LGBTQ college students whose lives were disrupted by COVID-19 a fair amount (aOR = 2.08; 95% CI = 1.11–3.89) or a great deal (aOR = 3.51; 95% CI = 1.83–6.71) compared to those whose lives were not disrupted much by COVID-19. Finally, about 38% of LGBTQ college students indicated concerns about their personal safety. After controlling for sociodemographic characteristics, safety concerns were significantly higher for bisexual students (aOR = 2.02; 95% CI = 1.05–3.90, compared to gay/lesbian individuals), students reporting “other” sexual identities (aOR = 3.14; 95% CI = 1.30–7.59, compared to gay/lesbian students), transgender students (aOR = 2.33; 95% CI = 1.02–5.32, compared to cisgender men), and LGBTQ students who were very concerned about the COVID-19 pandemic (aOR = 3.06; 95% CI = 2.07–4.53), compared to those not concerned about the pandemic).

**Table 5:** Prevalence and adjusted odds ratios of COVID-19 related vulnerabilities among LGBTQ college students, 18 years and older

	**Back in the Closet**		**Concerned About Personal Finances**		**Concerned About Personal Safety**	
	Prevalence (%)	aOR (95% CI)	Prevalence (%)	aOR (95% CI)	Prevalence (%)	aOR (95% CI)
**All Sampled LGBTQ College Students**	25.3	N/A	39.9	N/A	38.3	N/A
**Sexual Orientation**						
Gay/Lesbian	22.7	1.00 [Reference]	35.5	1.00 [Reference]	28.2	1.00 [Reference]
Bisexual	30.3	0.70 (0.31–1.58)	37.1	0.95 (0.51–1.78)	42.3	2.02 (1.05–3.90)**
Queer	25.4	0.91 (0.42–1.98)	37.7	0.95 (0.53–1.70)	37.7	1.41 (0.77–2.59)
Pansexual	19.2	0.44 (0.17–1.12)	49.4	1.55 (0.81–2.98)	44.3	1.72 (0.88–3.37)
Asexual	29.3	0.91 (0.37–2.25)	41.9	1.27 (0.62–2.61)	38.7	1.54 (0.73–3.22)
Questioning	22.9	0.46 (0.15–1.41)	42.1	1.31 (0.58–2.96)	36.8	1.19 (0.50–2.83)
Other	31.0	0.89 (0.25–3.10)	44.4	1.68 (0.72–3.91)	52.8	3.14 (1.30–7.59)**
**Gender Identity**						
Cisgender Man	14.1	1.00 [Reference]	30.2	1.00 [Reference]	26.0	1.00 [Reference]
Cisgender Woman	28.1	1.38 (0.61–3.14)	42.0	1.46 (0.81–2.61)	35.5	1.07 (0.58–2.00)
Transgender	13.0	0.68 (0.20–2.32)	46.8	1.43 (0.65–3.16)	48.9	2.33 (1.02–5.32)**
Non-Binary	34.4	1.77 (0.65–4.80)	33.9	0.94 (0.44–2.02)	49.2	1.74 (0.81–3.75)
Gender Non-Conforming	31.0	2.03 (0.58–7.07)	31.0	0.77 (0.29–2.08)	44.8	1.44 (0.54–3.81)
Agender	52.4	5.27 (1.37–20.26)**	50.0	1.65 (0.58–4.69)	50.0	1.80 (0.62–5.26)
Genderqueer	18.4	1.03 (0.33–3.24)	58.5	2.85 (1.30–6.22)**	45.3	1.88 (0.84–4.24)
Other	28.6	1.77 (0.42–7.35)	21.7	0.44 (0.14–1.44)	30.4	1.02 (0.33–3.18)
**Age, in years**						
18–20	36.7	6.83 (2.74–17.03)**	39.2	0.91 (0.52–1.58)	40.1	1.41 (0.79–2.53)
21–24	23.8	3.22 (1.29–8.04)**	42.2	1.07 (0.61–1.86)	37.9	1.24 (0.69–2.23)
25–29	8.2	1.00 [Reference]	39.1	1.00 [Reference]	31.0	1.00 [Reference]
≥ 30	5.6	0.53 (0.09–3.00)	33.3	0.66 (0.27–1.59)	47.2	1.63 (0.68–3.91)
**Race/Ethnicity**						
White	21.2	1.00 [Reference]	39.3	1.00 [Reference]	34.5	1.00 [Reference]
Black or African American	37.0	1.53 (0.67–3.50)	46.2	1.32 (0.69–2.53)	51.9	1.84 (0.95–3.57)
Hispanic/Latinx	22.7	0.74 (0.34–1.58)	46.6	1.34 (0.77–2.33)	43.8	1.48 (0.83–2.61)
Asian or Pacific Islander	40.0	1.28 (0.65–2.53)	31.1	0.58 (0.32–1.04)	43.2	1.45 (0.81–2.58)
Other	25.0	0.94 (0.21–4.19)	46.2	1.70 (0.51–5.67)	30.8	0.66 (0.18–2.50)
**Life Disrupted by COVID-19**						
Not at all or not much	13.2	1.00 [Reference]	22.7	1.00 [Reference]	32.0	1.00 [Reference]
A fair amount	24.8	1.45 (0.61–3.42)	37.2	2.08 (1.11–3.89)**	35.0	0.91 (0.50–1.65)
A great deal	30.1	2.09 (0.86–5.04)	49.5	3.51 (1.83–6.71)**	44.9	1.20 (0.65–2.21)
**Concerned About COVID-19**						
Not concerned or moderately concerned	25.4	1.00 [Reference]	40.4	1.00 [Reference]	25.4	1.00 [Reference]
Very or extremely concerned	25.3	1.00 (0.61–1.64)	39.5	0.86 (0.60–1.25)	49.4	3.06 (2.07–4.53)**
**Participant, family or friend tested positive for COVID-19**						
No	24.1	1.00 [Reference]	38.1	1.00 [Reference]	37.0	1.00 [Reference]
Yes	30.9	1.22 (0.67–2.22)	48.5	1.42 (0.89–2.27)	44.6	1.33 (0.82–2.17)
**Immediately Family Supportive of Participant's LGBTQ Identity**						
Supportive	6.4	1.00 [Reference]	34.1	1.00 [Reference]	32.3	1.00 [Reference]
Neither supportive nor unsupportive	25.0	4.39 (1.98–9.72)**	40.7	1.36 (0.79–2.35)	36.3	1.10 (0.62–1.94)
Unsupportive or family does not know	43.8	11.16 (5.76–21.63)**	45.0	1.49 (0.98–2.26)	44.6	1.53 (0.99–2.35)
**Feels Supported by their College or University LGBTQ Office**						
Yes	27.1	1.00 [Reference]	37.1	1.00 [Reference]	37.5	1.00 [Reference]
No, not much or no support	26.8	0.97 (0.56–1.69)	46.6	1.33 (0.87–2.01)	43.8	1.42 (0.93–2.19)
No opinion or missing data	18.9	0.64 (0.32–1.27)	36.3	0.86 (0.51–1.43)	31.9	0.75 (0.44–1.29)

Source: Online sample of LGBTQ identified college students from April 24—June 5, 2020. Notes: aORs were obtained from multivariable logistic regression models adjusting for all the covariates listed in the table. **p < 0.05

## Discussion

This study provides a more in-depth examination of LGBTQ college student health and wellbeing compared to our previously reported analysis [13]. The present study found that LGBTQ college students were experiencing substantial distress, barriers to medical and mental health care, and concerns about their personal finances, safety, and ability to fully express their LGBTQ identities as colleges and universities transitioned to remote and virtual learning in the spring of 2020. Upwards of 25 million college students were experiencing disrupted learning and additional stress at the onset of the COVID-19 pandemic, and LGBTQ college students may have been especially vulnerable. Although this study did not make direct comparisons to non-LGBTQ students, other research has suggested that LGBTQ college students reported elevated levels of psychological distress before and during the COVID-19 pandemic compared to non-LGBTQ students [20]. Moreover, this study highlighted experiences unique to members of the LGBTQ community that may be missed in other population-based research, such as going back into the closet at home and worrying about seeking care due to one’s sexual orientation or gender identity.

We found consistent health vulnerabilities among a subset of LGBTQ college students. For instance, Hispanic/Latinx students were more likely to report poor/fair self-rated health, elevated levels of unmet medical care needs, and concerns about seeking medical care because of their LGBTQ status compared to White LGBTQ students. This finding should serve as a reminder that intersectional approaches to research, policy, and practice remain critical. Some Hispanic/Latinx LGBTQ college students may experience distress when coming out to their families due to *machismo*, familism, or religious beliefs of the family [27]. Therefore, social support providers, college counselors, and mental health providers should continue to provide cross-cultural assessments and tools for Hispanic/Latinx LGBTQ college students to thrive [28].

We also found that, compared to cisgender men, transgender and gender diverse (including non-binary, agender, and genderqueer) students were more likely to report poor/fair self-rated health, personal safety concerns, unmet medical care needs, and concerns about seeking mental health care because of their gender identities. These findings may exacerbate underlying mental health disparities faced by gender diverse adolescents and young adults [29]. Public health campaigns and social supports should engage with and provide outreach to transgender and gender diverse students to support their health and wellbeing. Relatedly, medical institutions and professional psychology training programs should review their education and training offerings in LGBTQ health to ensure health care providers receive training on the unique health needs of transgender populations in the United States — either through education for students and trainees on cultural competencies and gender-affirming care or through continuing education requirements for practicing providers.

LGBTQ students who did not feel supported by their college or university also reported significantly greater levels of unmet medical care and mental health care needs. Colleges should consider mitigating the impact of hunger, homelessness, financial challenges, and lack of access to the internet and/or technology – issues which impact all students but may be particularly pronounced for LGBTQ students during and after the COVID-19 pandemic. Some research suggests that novel zoonotic viruses will be more common as global temperatures continue to rise [30]. Thus, investing in the adequate resources now is essential for alleviating the effects of future pandemics.

Meanwhile, many residential students rely on the student health centers and campus resources while attending college. Indeed, evidence suggests that LGBTQ students are more likely to seek on-campus mental health services compared to non-LGBTQ students [31]. Colleges and universities should consider providing telehealth services when possible and referrals to inclusive licensed professionals when students are enrolled remotely or visiting family during holidays, summer vacation, and fall/spring breaks.

LGBTQ college students with families that were unsupportive or unaware of the participant’s LGBTQ status reported poor/fair health, dissatisfaction with life, unmet mental health care needs, and having to go back into the closet during the COVID-19 pandemic. Other studies have also found that returning to parental homes during COVID-19 was associated with LGBTQ identity concealment and bans on seeing romantic or sexual partners [22, 23]. Colleges should provide resources (e.g., counseling, information, and mediation) for students, parents, and families experiencing difficulties related to gender and sexuality. Moreover, students and families should be introduced to LGBTQ resources (among other identity-based resources) during visitation days and orientation. Finally, health care providers (especially those treating adolescents and young adults) should consider clinical encounters as educational opportunities to inform parents and other family members on the scientific consensus of evidence-based clinical guidelines for the affirmation of gender identity and sexual orientation.

### Limitations

Limitations of this study include its nonrandom-based sampling that may not be generalizable to the entire LGBTQ college student population. Moreover, we were limited by the sample sizes based on volunteered participation. Thus, small sample sizes prevented us from exploring specific identities with fewer participants (e.g., polysexual, genderfluid, or intersectional analyses by more detailed racial and ethnic identities). Our study was also limited by selection bias and may be missing LGBTQ college students who were unhoused, lacking internet access, or were not comfortable responding to an LGBTQ-focused online survey. Our targeted advertisements may have also missed students who were avoiding or unable to access social media because of privacy concerns, stressful news coverage, or traumatic experiences of returning home. Finally, our sample only included LGBTQ college students in the United States, and data from non-LGBTQ college students were not collected. Future studies should leverage population-based samples, when available, to address these concerns.

## Conclusion

While the case count and death toll due to COVID-19 rises as we transition from a COVID-19 pandemic to endemic infection, the virus has exacerbated many of the contextual issues that already impacted LGBTQ college students before the pandemic took hold. Several studies have documented the adverse mental and behavioral health impacts of the pandemic on LGBTQ college students. We provide more evidence to document the experiences of LGBTQ college students at the start of the pandemic with a focus on self-rated health, personal safety, and access to care, finding that large proportions of LGBTQ college students were dissatisfied with life and concerned about their mental health, finances, and safety, and faced unsupportive families and unmet medical and mental health care needs. Even within this group, there are important intragroup differences to recognize. LGBTQ students of color, transgender students, and gender-diverse students have a greater likelihood of enduring worse physical health, mental health, and access to care. While the long-term effects of the pandemic on LGBTQ students are still unknown, findings from the earliest days of the pandemic can inform best practices for achieving LGBTQ health equity among young adults enrolled in colleges and universities during future public health emergencies. Ensuring everyone’s safety and wellbeing during and following the pandemic will have lasting effects on reducing health disparities.

## Supplementary Information

**Additional file 1.**

## Data Availability

The datasets generated during and/or analyzed during the current study are available from the corresponding author on reasonable request.

## Abbreviations

COVID-19: Coronavirus disease
LGBTQ: Lesbian, gay, bisexual, transgender, queer, and questioning
Aor: Adjusted odds ratios
CI: Confidence intervals

## Declarations

### Questionnaire

The questionnaire was developed for this study and is uploaded as a supplementary file.
