# Supplementary material for: LGBTQ College student health and wellbeing at the onset of the pandemic: additional evidence and lessons learned from COVID-19
Source: BMC Public Health. 2023 May 26;23:967. doi: 10.1186/s12889-023-15909-z (PMC10214329; doi:10.1186/s12889-023-15909-z)
Supplement: Supplementary file 1 — Additional file 1. [file 12889_2023_15909_MOESM1_ESM.pdf]

LGBTQ College Health Survey During COVID-19

Record ID ____________________

You are invited to participate in a research study to better understand how the coronavirus (COVID-19) pandemic has affected the health and wellbeing of LGBTQ college students. You will be asked questions about your current health status, living arrangements, health behaviors, access to medical care, and the actions you have taken thus far in response to the coronavirus outbreak.
Key information about this study
The purpose of this study is to better understand how the coronavirus (COVID-19) pandemic has affected the health and wellbeing of LGBTQ college students (18 years and older).
Participation in this study will involve the completion of one online survey that will take about 10 to 15 minutes. The survey will ask you about your current health status, living arrangements, health behaviors, access to medical care, and the actions you have taken during the coronavirus outbreak.
Participation in the study is completely voluntary and involves the completion of one online survey that will take approximately 10 to 15 minutes. You may sign up at the end of the survey to voluntarily join an online focus group, and we will provide you additional details for participating in online focus groups. The submission of your name and email address will be stored separately from your survey responses. You may choose not to share your email address and still participate in the survey.
The risks associated with this study are minimal and include potential loss of confidentiality and psychological distress due to answering questions about the COVID-19 outbreak. We cannot and do not guarantee or promise that you will receive any direct benefits from this study, though your responses may be used to improve the LGBTQ community response to the COVID-19 outbreak.
All responses will remain anonymous and confidential. The only personal information asked will be at the end of the survey if you would like to follow-up and participate in online focus groups. If any of the research questions make you uncomfortable, you are free to decline to answer any questions you don't wish to answer, or to stop participating at any time.
Contact Information
If you should have any questions about this research study, payments, or possibly injury, please feel free to contact Dr. Gilbert Gonzales via email at gilbert.gonzales@vanderbilt.edu or by phone at 615-343-6958.
For additional information about giving consent or your rights as a participant in this study, to discuss problems, concerns, and questions, or to offer input, please feel free to contact the Institutional Review Board Office at (615) 322-2918 or toll free at (866) 224-8273.
STATEMENT BY PERSON AGREEING TO PARTICIPATE IN THIS STUDY
I have read this informed consent document. I freely and voluntarily choose to participate.

◯ Yes, I would like to participate
◯ No, I do not want to participate

Age Confirmation

Are you 18 or older?

◯ Yes
◯ No

Demographic Information and General Questions

How old are you? ____________________

What is your current gender identity? (Please select all that apply)

- ☐ Male/Man
- ☐ Female/Woman
- ☐ Transgender
- ☐ Non-Binary
- ☐ Gender Non-Conforming
- ☐ Agender
- ☐ Genderqueer
- ☐ An identity not listed above (Please specify)

Please state your gender identity here. ____________________________

What sex were you assigned at birth, meaning on your original birth certificate?

- ○ Male
- ○ Female
- ○ Prefer not to say

Do you consider yourself to be: (Please select all that apply)

- ☐ Heterosexual or straight
- ☐ Lesbian
- ☐ Gay
- ☐ Bisexual
- ☐ Queer
- ☐ Pansexual
- ☐ Asexual
- ☐ Questioning or unsure
- ☐ A sexual orientation not listed above (Please specify)

Please state your sexual orientation here. ____________________________

Which of the following would you say is your race/ethnicity? (Please select all that apply)

- ☐ White
- ☐ Black or African Americans
- ☐ Hispanic or Latino/a
- ☐ American Indian or Alaska Native
- ☐ Asian
- ☐ Pacific Islander
- ☐ A race/ethnicity not listed above (Please specify)

Please state your race/ethnicity here. ____________________________

Were you enrolled as a community college, college, or university student as of March 1, 2020?

- ○ Yes, full-time student
- ○ Yes, part-time student
- ○ No, not enrolled

Would you be willing to provide us the name of your community college, college, or university? (All responses will remain anonymous and will not be shared with your institution; this is only for categorization purposes)

- ○ Yes
- ○ No

What is the name of your community college, college, or university (again, all responses will remain anonymous and will not be shared with your institution; this is only for categorization purposes)?

____

Which of the following do you attend?	◯ Community college or technical school<br>◯ Public university or college<br>◯ Private university or college
Approximately how many students attend your college or university?	◯ Less than 5,000<br>◯ 5,000-15,000<br>◯ 15,000-30,000<br>◯ More than 30,000

As of March 1,2020, what was your classification at your college or university?

◯ First-year undergraduate
◯ Second-year undergraduate
◯ Third-year undergraduate
◯ Fourth-year undergraduate
◯ Fifth-year+ undergraduate
◯ Master's student
◯ Doctoral student
◯ Other (Please specify)

If other, please specify here.	__________________________________
Were you living on-campus or off-campus for the 2019-2020 academic year?	◯ On-campus<br>◯ Off-campus<br>◯ I don't know
Were you required to vacate your on-campus housing early due to the coronavirus (COVID-19) outbreak?	◯ Yes<br>◯ No
If you were required to vacate your on-campus housing, how many days were you given to leave? (Please answer in whole numbers only)	__________________________________

If you were required to vacate your on-campus housing, where are you currently living?

◯ In a house, apartment, or condo alone
◯ With parents or family I grew up with
◯ With a host who is not related to me
◯ With a partner or spouse
◯ Temporarily with friends or relatives
◯ In a hotel or motel
◯ In transitional housing/halfway house
◯ In a homeless shelter
◯ I do not have housing. I am living on the street or a place that is NOT a house, apartment, shelter, or other housing.

How many other adults (18+ years old) are you currently living with?	__________________________________
How many children (17 years and younger) are in your home or living with you?	__________________________________
Are you classified by your community college, college, or university as an international student?	◯ Yes<br>◯ No

What was your employment status prior to the coronavirus (COVID-19) outbreak?

☐ Full-time employment
☐ Part-time employment, off-campus job
☐ Part-time employment, on-campus job
☐ Was not working but was looking for work
☐ Was not working and was not looking for work

Have you received any of the following for the 2019-2020 academic year? (Please select all that apply)

☐ Merit-based scholarships
☐ Federal Pell Grant
☐ Federal Stafford Loan
☐ Federal Work Study
☐ Hardship funding or emergency aid in response to the coronavirus outbreak
☐ Other need-based funding
☐ Do not know
☐ None of these

What is the primary source of your health care coverage?

◯ My parent's health insurance plan
◯ Student health insurance offered by my university
◯ Private health insurance I obtained on my own
◯ Medicaid
◯ Medicare
◯ TRICARE/CHAMPUS, VA, or Military
◯ Alaska Native, Indian Health Service, Tribal Health Services
◯ Some other source
◯ I am uninsured
◯ I don't know the answer to this question

Are you currently able to receive medical care if needed?

◯ Yes
◯ No, because of cost
◯ No, because of 'stay at home order' restrictions
◯ No, because of other reasons

If other, please state here. ______________________________

Are you currently able to receive mental health care if needed?

◯ Yes
◯ No, because of cost
◯ No, because of 'stay at home order' restrictions
◯ No, because of other reasons

If other, please state here. ______________________________

What is your state/territory of residence? (Please choose N/A if an international student)

◯ Alabama
◯ Alaska
◯ Arizona
◯ Arkansas
◯ California
◯ Colorado
◯ Connecticut
◯ Delaware
◯ Florida
◯ Georgia
◯ Hawaii
◯ Idaho
◯ Illinois
◯ Indiana
◯ Iowa
◯ Kansas
◯ Kentucky
◯ Louisiana
◯ Maine
◯ Maryland
◯ Massachusetts
◯ Michigan
◯ Minnesota
◯ Mississippi
◯ Missouri
◯ Montana
◯ Nebraska
◯ Nevada
◯ New Hampshire
◯ New Jersey
◯ New Mexico
◯ New York
◯ North Carolina
◯ North Dakota
◯ Ohio
◯ Oklahoma
◯ Oregon
◯ Pennsylvania
◯ Puerto Rico
◯ Rhode Island
◯ South Carolina
◯ South Dakota
◯ Tennessee
◯ Texas
◯ Utah
◯ Vermont
◯ Virginia
◯ Washington
◯ West Virginia
◯ Wisconsin
◯ Wyoming
◯ N/A

How would you describe the social climate of your home community?

◯ Conservative
◯ Moderate
◯ Progressive
◯ Unsure

Is the college or university that you attend in your state of residence?

◯ Yes
◯ No

If different from your state of residence, in which state do you attend school?

◯ Alabama
◯ Alaska
◯ Arizona
◯ Arkansas
◯ California
◯ Colorado
◯ Connecticut
◯ Delaware
◯ Florida
◯ Georgia
◯ Hawaii
◯ Idaho
◯ Illinois
◯ Indiana
◯ Iowa
◯ Kansas
◯ Kentucky
◯ Louisiana
◯ Maine
◯ Maryland
◯ Massachusetts
◯ Michigan
◯ Minnesota
◯ Mississippi
◯ Missouri
◯ Montana
◯ Nebraska
◯ Nevada
◯ New Hampshire
◯ New Jersey
◯ New Mexico
◯ New York
◯ North Carolina
◯ North Dakota
◯ Ohio
◯ Oklahoma
◯ Oregon
◯ Pennsylvania
◯ Rhode Island
◯ South Carolina
◯ South Dakota
◯ Tennessee
◯ Texas
◯ Utah
◯ Vermont
◯ Virginia
◯ Washington
◯ West Virginia
◯ Wisconsin
◯ Wyoming
◯ N/A

Is the social climate of the place (city, town, etc.) in which you attend school different from your home community?

◯ Yes, the social climate of the city or town where I attend school is more progressive
◯ Yes, the social climate of the city or town where I attend school is more conservative
◯ Not much different

Is the social climate of your school community different from your home community?

◯ Yes, the social climate of my school community is more progressive
◯ Yes, the social climate of my school community is more conservative
◯ Not much different

General Health Questions

This section is going to focus on your health. Questions will address your general health, as well as your health over the last 30 days and 2 weeks.

In general, would you say that your health is...

◯ Excellent
◯ Very Good
◯ Good
◯ Fair
◯ Poor

Now thinking about your physical health, which includes physical illness and injury.

For how many days during the past 30 days was your physical health not good? __________________________________

Now thinking about your mental health, which includes stress, depression, and problems with emotions.

For how many days during the past 30 days was your mental health not good? __________________________________

During the past 30 days, how many days did you have at least one drink of any alcoholic beverage such as beer, wine, a malt beverage or liquor? __________________________________

During the past 30 days, on the days that you drank, approximately how many drinks did you have (one drink is equivalent to a 12-ounce beer, a 5-ounce glass of wine, or one shot of liquor)? __________________________________

These questions are going to address experiences over the last two weeks (if you are using a cell phone or tablet to complete this survey, it may be helpful to tilt your screen).

	Never	For several days	For more than half the days	Nearly every day
Over the last 2 weeks, how often have you been bothered by having little interest or pleasure in doing things? Would you say this happens...	◯	◯	◯	◯

Over the last 2 weeks, how often have you been bothered by feeling down, depressed or hopeless? Would you say this happens...	○	○	○	○
Over the last 2 weeks, how often have you been bothered by feeling nervous, anxious or on edge? Would you say this happens...	○	○	○	○
Over the last 2 weeks, how often have you been bothered by not being able to stop or control worrying? Would you say this happens...	○	○	○	○
Over the last 2 weeks, how often have you participated in any elective physical activities such as running, biking, weight training, or walking?	○	○	○	○
Over the last 2 weeks, how often have you had trouble falling asleep or staying asleep?	○	○	○	○

How often do you get the social and emotional support you need?

○ Always
○ Usually
○ Sometimes
○ Rarely
○ Never

In general, how satisfied are you with your life?

○ Very satisfied
○ Satisfied
○ Dissatisfied
○ Very dissatisfied

Coronavirus (COVID-19) Outbreak Questions

In this section, we will be asking questions regarding the effects of the coronavirus (COVID-19) outbreak on your life and the lives of those that you know.

To what extent has your own life been affected or disrupted by the coronavirus (COVID-19) outbreak?

○ A great deal
○ A fair amount
○ Not much
○ Not at all
○ No opinion

In the past 30 days weeks, have you or a close friend or loved one tested positive for coronavirus (COVID-19)?

☐ Yes, I have tested positive for coronavirus (COVID-19)
☐ Yes, a close friend or loved one has tested positive for coronavirus (COVID-19)
☐ No, neither I nor a close friend or loved one has tested positive for coronavirus (COVID-19)

How concerned do you feel about coronavirus (COVID-19)?

◯ Not at all concerned
◯ A little concerned
◯ Moderately concerned
◯ Very concerned
◯ Extremely concerned

In an effort to reduce the spread of coronavirus (COVID-19), many are practicing social distancing and self-isolation. Self-isolation is the act of staying away from situations where you may be in close contact with others, such as social gatherings, work, school, faith-based gatherings, sports gatherings, restaurants, and other public gatherings.

To what extent are you self-isolating?

◯ All of the time. I am staying home nearly all of the time.
◯ Most of the time. I only leave my home to buy food and other essentials.
◯ Some of the time. I have reduced the amount of times I am in public spaces, social gatherings or at work.
◯ None of the time. I am doing everything I normally do.

For how many days have you been self-isolating?
(Please answer in whole numbers only) ________________________________

What are the reasons you are not self-isolating more? (Please select all that apply)

☐ I cannot afford to miss work
☐ I do not have the option to work from home
☐ I do not have my own space to self-isolate in
☐ I do not think self-isolation is effective in preventing the spread of COVID-19
☐ I have to attend class in-person
☐ I have to care for others outside of my home
☐ I do not believe I am at high risk for COVID-19
☐ I am a healthcare worker/first responder
☐ Other (Please specify below)

If other, please specify here.

____

During the past 30 days, how many days did you cut the size of your meals or skip meals because you did not have the money or means to acquire food?

____

Have you noticed any of the following changes in your sleep? (Please select all that apply)

☐ I am sleeping longer
☐ I am sleeping less
☐ I have altered the time of day that I sleep
☐ Stayed about the same or no change

The coronavirus (COVID-19) outbreak is a threat to my physical health. Do you...?

◯ Strongly agree
◯ Agree
◯ Not sure
◯ Disagree
◯ Strongly disagree

The coronavirus (COVID-19) outbreak is a threat to my mental health. Do you...?

◯ Strongly agree
◯ Agree
◯ Not sure
◯ Disagree
◯ Strongly disagree

Since the coronavirus (COVID-19) outbreak, do you find yourself more likely to do any of the following? (Please select all that apply)

☐ Consume alcohol
☐ Smoke cigarettes, e-cigarettes, or Juul
☐ Use drugs
☐ No change

On average, how supportive are members of your immediate family (parents, sisters, brothers, etc.) of your sexual orientation or gender identity?

◯ Very supportive
◯ Supportive
◯ Neither supportive or unsupportive
◯ Unsupportive
◯ Very unsupportive
◯ My immediate family members do not know about my sexual orientation or gender identity

To what extent are you out of the closet to the following groups of people in your life:

	Out to all	Out to most	Out to some	Out to none	Not applicable	Don't know
...immediate family (parents, sisters, brothers, etc)?	◯	◯	◯	◯	◯	◯
...extended family?	◯	◯	◯	◯	◯	◯
...friends you grew up with?	◯	◯	◯	◯	◯	◯
...friends from college?	◯	◯	◯	◯	◯	◯
...co-workers?	◯	◯	◯	◯	◯	◯
...health care providers?	◯	◯	◯	◯	◯	◯

Have you gone back "in the closet" as a result of the coronavirus (COVID-19) outbreak?

◯ Yes, I've gone back in the closet to some extent
◯ No, my "outness" has not changed
◯ Don't know

If yes, please mark all groups for whom you have gone back "in the closet" as a result of the coronavirus (COVID-19)?

☐ Immediate family (parents, sisters, brothers, etc)
☐ Extended family
☐ Friends you grew up with
☐ Friends from college
☐ Co-workers
☐ Health care providers
☐ Other people (please specify)

If other people, please specify here.

____

Since the coronavirus (COVID-19) outbreak, how has the amount of social support you seek changed?

◯ Seek more social support from my friends
◯ Seek more social support from my family
◯ Seek more social support from a combination of friends and family
◯ No real change
◯ Seek less social support from my friends
◯ Seek less social support from my family
◯ Seek less social support from a combination of friends and family

To what extent do you worry about seeking care due to your LGBTQ status should you become infected with coronavirus (COVID-19)?

◯ A great deal
◯ A fair amount
◯ Not much
◯ Not at all
◯ No opinion

Has the coronavirus (COVID-19) outbreak caused any personal financial hardship?

◯ Yes
◯ No

If yes, how has the coronavirus (COVID-19) outbreak caused any personal financial hardship? (Please select all that apply)

☐ I lost my job
☐ I received a pay cut
☐ I lost money for dining at my college or university that was not refunded to me
☐ I lost money for housing at my college or university that was not refunded to me
☐ I have yet to receive any refund from my college or university
☐ I am having trouble paying for basic needs (food, housing, clothing)

Has the coronavirus (COVID-19) outbreak put you in a position where you feel unsafe?

◯ Yes
◯ No

To what extent are you concerned with how the coronavirus (COVID-19) outbreak may be impacting your LGBTQ peers?

◯ A great deal
◯ A fair amount
◯ Not much
◯ Not at all
◯ No opinion

	A great deal	A fair amount	Not much	Not at all	No opinion
To what extent do you still feel supported by the LGBTQ center at your college/university or by other campus resources available to LGBTQ students?	◯	◯	◯	◯	◯
To what extent do you still feel supported by your LGBTQ peers from your college/university?	◯	◯	◯	◯	◯
To what extent do you feel supported by the LGBTQ community where you are now living?	◯	◯	◯	◯	◯

If your school decided to hold classes online in the fall semester of 2020, would you be concerned about: (Please select all that apply)

☐ Your ability to study regularly
☐ Your ability to continue to attend school
☐ Your ability to stay where you are currently living
☐ Your ability to pay for basic needs like food, housing, or clothing
☐ Your ability to be open about your self-expression or to feel accepted
☐ Something else
☐ None of these
☐ Not applicable

Open-Ended Questions

In this section, you will respond to two short-answer questions regarding your experience during the COVID-19 Pandemic.

In light of the coronavirus (COVID-19) outbreak, please describe the ways you have felt challenged as an LGBTQ individual.

Considering the coronavirus (COVID-19) outbreak, please describe the ways in which your life has been disrupted or has changed.

What additional resources or services, if offered by your college/university, would you most benefit from during uncertain times such as the coronavirus (COVID-19) outbreak?

Thank you!

We invite you to continue on with our study by joining our virtual focus group sessions. During these focus groups, we will facilitate further discussion on the challenges you may have encountered or may be encountering during the coronavirus (COVID-19) outbreak. If you decide to participate, you will be grouped with individuals with a similar sexual orientation or gender identity. We hope that this may provide you with an opportunity to draw strength from the LGBTQ community during this incredibly difficult time.

Would you like to participate in our voluntary virtual focus group?

◯ Yes
◯ No

Focus Group Contact Information

Please provide email here. __________________________________
